# Pyogenic liver abscess caused by Gemella morbillorum

**Published:** 2014-06-30

**Authors:** Paolo Borro, Alessandro Sumberaz, Gianni Testino

**Affiliations:** IRCCS Azienda Ospedaliera Universitaria San Martino-IST, Genova, Italy

**Keywords:** Abscess, liver, Gemella morbillorum

## Abstract

Even though *Gemella morbillorum* infection (GMI) is rare in humans, it may nevertheless, cause endocarditis, meningitis, brain abscess, pleural empyema, nephritis, mediastinitis, and occasionally, liver abscess. We are describing the case of a 64-years-old Caucasian male admitted with fever and abdominal pain. Laboratory parameters revealed inflammation signs, and instrumental examinations showed the presence of diverticula in the ascending colon. Abdominal ultrasound (US) and computer tomography (CT) showed two focal lesions in the right liver lobe. One had the characteristics of a simple cyst; the second was hypoechoic with a low density area, possibly containing necrotic material. US-guided needle biopsy was found negative for neoplastic cells, showing purulent infiltrate. Pus culture was found positive for GMI. Systemic antibiotic therapy coupled with repeated US-guided needle aspiration, induced the resolution of the hepatic abscess. Few cases have been reported of hepatic abscess caused by GMI in immunocompetent non-cirrhotic subjects.

## Abstract (es)

A pesar de que la infección por *Gemella morbillorum *(GMI por el término en inglés) es poco común en seres humanos, puede causar endocarditis, meningitis, absceso cerebral, empiema pleural, nefritis, mediastinitis y en ocasiones, absceso hepático. Describimos el caso de un hombre caucásico de 64 años que ingresó con fiebre y dolor abdominal. Los parámetros de laboratorio revelaron signos de inflamación y los exámenes mostraron la presencia de divertículos en el colon ascendente. La ecografía abdominal (US) y la tomografía computarizada (CT) mostró dos lesiones focales en el lóbulo hepático derecho. Una presentó las características de un quiste simple; la segunda fue hipoecóica con una zona de baja densidad, que posiblemente contenía material necrótico. Biopsia con aguja guiada por US dio un resultado negativo para células neoplásicas, mostrando infiltrado purulento. Cultivo de pus fue encontrado positivo para GMI. Una terapia con antibióticos sistémicos, junto con aspiración repetida con aguja guiada por US indujo a la resolución del absceso hepático. Pocos casos se han reportado de absceso hepático causado por GMI en sujetos inmunocompetentes no cirróticos.

## Introduction

In immunocompetent patients, pyogenic hepatic abscess (PHA) is often cryptogenetic or secondary to ascending colangitis, diverticolitis or appendicitis. Moreover, the risk of developing a hepatic abscess increases when chronic diseases such as diabetes, cirrhosis, neoplasia, or liver transplant are present.

Commonly, the process is caused by flogistic focuses in the colorectum mucosa, pancreas, cholecyst, or the biliary tree. From these regions the microorganism spreads via bile or portal system into the liver, causing an abscess. The most common pathogens involved in this process are *Escherichia coli, Klebsiella pneumoniae*, Bacteroides, Enterococci, Streptococci, Staphylococci, and -occasionally- *Salmonella typhi*. Despite the fact that diagnosis of a hepatic abscess is easy using abdominal ultrasound (US) and/or computer tomography (CT), the differential diagnosis between primitive or secondary neoplasm may be difficult.

The diagnosis of hepatic abscess is carried out by using the US-guided needle biopsy; whereas, treatment is based on both US-guided needle aspiration and systemic antibiotic therapy.

We are describing a case of hepatic abscess caused by *Gemella morbillorum* infection (GMI), a saprophyte microaerophilic Gram-positive coccus of gastrointestinal, urogenital and respiratory mucosa, which eventually may cause endocarditis, meningitis, cerebral abscess, pleural empyema, nephritis, and mediastinitis 1 ^,^ 2.

## Case report

On March 2010, a 64-year-old immunocompetent man was hospitalized for hyperpyrexia (38-39° C) and epigastrium pain, which had started suddenly one week earlier. He was not taking any medications, was not abusing alcohol and had not been abroad. His past medical history was clear. Physical examination showed no skin rash, no abdominal tenderness; palpable liver, spleen, and abdominal mass were all absent. Defecation and diuresis were normal. Laboratory parameters revealed: WBC 14x10^3 ^cell/(L (neutrophils 87%); ESR 109 mm/h, CRP 33 mg/dL, fibrinogen 1,230 mg/dL. Alanine aminotransferase resulted 120 IU/L (n.v. 0-40 IU/L) and (-glutamyl transferase 200 IU/L (n.v. 8-61 IU/L); bilirubin was in the normal range.

**Figure 1.**
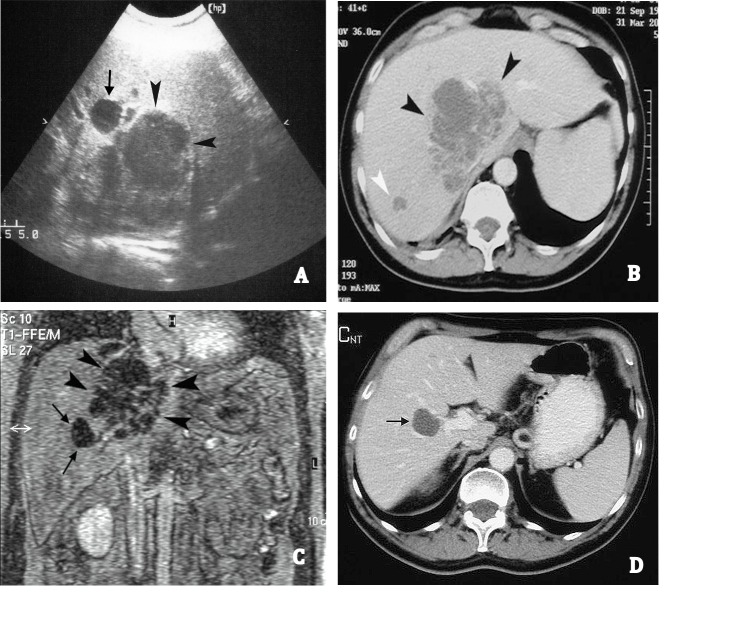
**A.** US shows a large hypoechoic lesion with ill-defined margins (black arrow heads) and an anechoic lesion (black arrow). **B.** Abdominal spiral CT reveals a hypointense area that was slightly enhanced in the periphery and in septations after contrast administration (black arrow heads) and a small lesion with similar density (white arrow head). **C.** MRI reveals a large sepimented lesion (black arrow heads) and a small hypointense lesion referable to a liver cyst (black arrows). **D.** A contrast-enhanced CT carried out 20 days after the discharge shows a hypointense lesion referred to a liver cyst (black arrow) and a complete resolution of the abscess.

Blood test was negative for HIV, HCV, HBV, and other hepatotropic viruses. Six haemocultures collected over two consecutive days, as well as chest radiography, resulted negative. Abdominal US revealed a liver lesion of approximately 10 cm in diameter, with irregular form and poorly defined margins, located in the lateral section of the right liver lobe, close to the vena cava. No intra-hepatic or extra-hepatic biliary dilatation was observed. The liver, spleen, pancreas, and kidneys were of normal size. Spiral CT confirmed the low-density liver area slightly enhanced at the early phase after injection of a contrast medium. Also, the T2-weighted magnetic resonance images (MRI) confirmed the mass with high signal intensity and with slight enhancement at the early phase after gadolinium administration. All three imaging techniques gave rise to the suspicion of a necrotic sepimented area or neoplastic liver lesion. Furthermore, two other small lesions with similar density to the above were identified in liver segments 5 and 7. An anechoic ovalar lesion, close to the largest lesion, referable to a simple liver cyst was also detected (Figs. 1A, B and C). 

Treatment with antibacterial (ceftriaxone 2 g bid iv + metronidazole 500 mg tid iv) and antifungal (itraconazole 200 mg iv) was started after hemocultures were collected. Fever responded poorly to paracetamol. Thoracic CT, gastroscopy and tumoral markers resulted negative; whereas, colonoscopy revealed diffuse large diverticula in the ascending colon.

A US-guided needle (Chiba 18 G) biopsy with aspiration of the liver lesion showed abundant (60 mL) purulent fluid which resulted positive for *Gemella morbillorum*. Focal necrosis with fibrosis, but no atypical cells, was observed in the examined sections.

Needle aspiration induced prompt resolution of the hyperpyrexia, but persistent irregular low fever continued.

On the basis of the antibiogram, therapy using teicoplanin (6 mg/kg/day 1 and then 3 mg/kg/day iv) plus imipenem (1.5 g/day iv) was replaced with the former therapy.

Ten days later, the US revealed a liver lesion of approximately 3 cm in diameter and the febricula was still present. Two other US-guided needle (Chiba 18 G) aspirations of the liver lesion collected 30 and 20 mL of pus, respectively, on day 20 and 30 and the fever disappeared. The flogistic parameters (ESR, CRP, etc.,) also returned to normal values, and the patient was discharged with instructions to continue antibiotic treatment for a total of 30 days. Abdominal US and CT carried out 20 days after discharge revealed complete resolution of the liver lesion. A follow up visit, 6 months after discharge, showed the patient in good health (Fig. 1D). 

## Discussion

Pyogenic hepatic abscess is rare in industrialized countries with an incidence of 11.8 (men), and 9.7 (women) cases per million. Recently, the trend was found slightly raised. When PHA occurs, the mortality rate is high, 26.9% after 30 days, and it can increase to 62.5% in patients affected by cirrhosis. The most common pathogens of hepatic abscess are *E. coli, Klebsiella pneumoniae*, Bacteroides, Enterococci, Streptococci, and Staphylococci. However, to our knowledge, few reports exist on PHA related to a GMI, summarized in Table 1. The liver is a possible -though rare- site for lesions from ascending colangitis, diverticulitis, or appendicitis related to portal venous drainage of visceral organs.

**Table 1.**
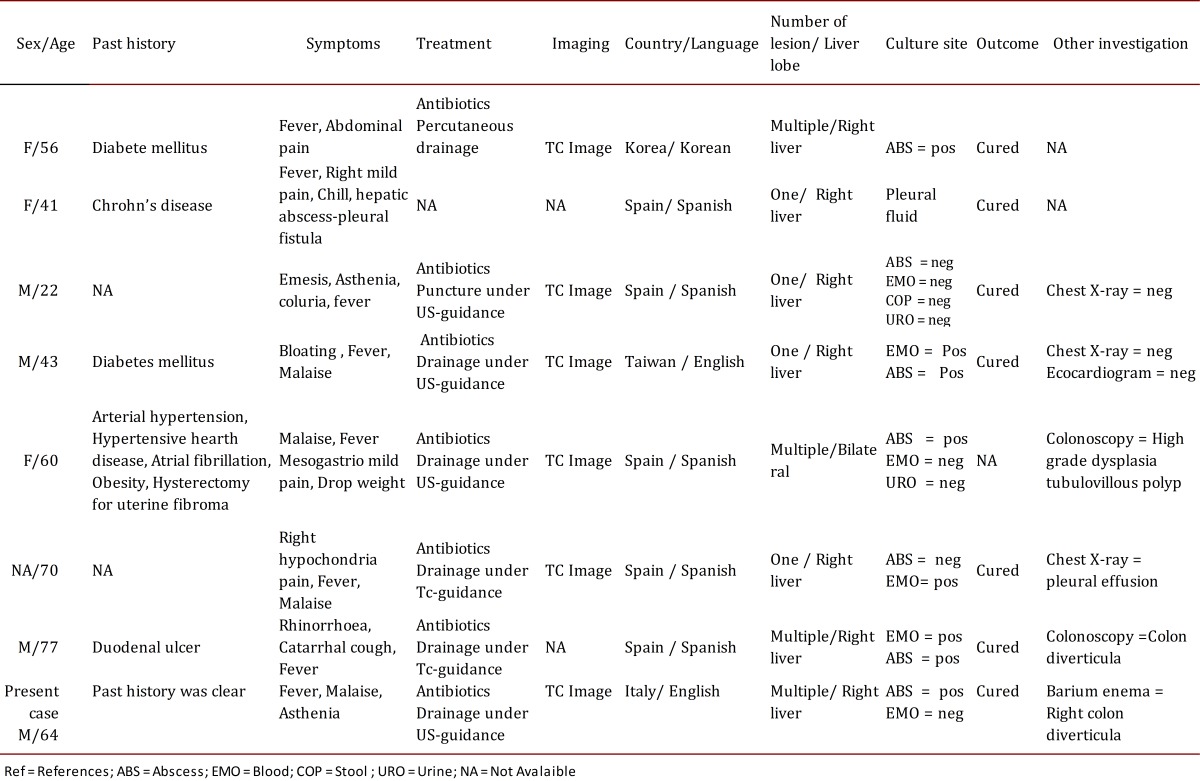
Published cases of liver abscess due to *Gemella morbillorum*

In our patient, fever, abdominal pain, leukocytosis, elevation of ESR and CRP gave rise to suspicion of infection. Both US and radiologic images show the presence in the side sector of the right liver lobe of an irregular lesion with poorly defined margins, and all the imaging techniques gave the suspicion of necrotic areas in an abscess or neoplasm liver lesion (primary or metastatic). Despite the elevated sensitivity of US, CT, and MRI, it is often difficult to make a differential diagnosis between a septic liver abscess and a primitive or secondary hepatic neoplasia.

The US-guided needle biopsy and aspiration excluded the presence of neoplastic cells and the purulent fluid resulted positive for *Gemella morbillorum*. *Gemella morbillorum* is a saprophyte ubiquitary gram+ coccus often present in the gastrointestinal, urogenital and respiratory mucosa. In this case the most probable source of GMI is in the large diverticula of the ascending colon, diagnosed via colonoscopy. Intestinal mucosal infection may then have spread to the liver through portal vein drainage 3 ^,^ 4. 

Percutaneous aspiration is the method of choice used to identify the pathogens (89.0%) and remove purulent infection. Ch Yu *et al*., reported 101 cases of liver abscess (98.4% pyogenic), 28.1% were multiple: the percentage of positive needle aspiration was 96.8 and the success rate was unrelated to the size or number of abscesses in a single patient. Moreover, Yu *et al*., reported that intermittent needle aspiration, when compared to continuous catheter drainage, was associated with higher treatment success rate, shorter duration of hospital stay, and lower mortality rate, although this did not reach statistical significance 5. 

The other two intrahepatic small "metastatic" abscessual lesions were resolved with antibiotic treatment without percutaneous aspiration. Antibiotic therapy resolves up to 80% of liver abscess and this, coupled with US-guided needle aspiration, yields virtually 100% success rate 6 ^,^ 7. 

## Conclusion

Pyogenic hepatic abscess is rare in immunocompetent and non-cirrhotic subjects. It is mostly secondary to ascending colangitis, diverticolitis or appendicitis and is caused by pathogens. In this clinical case, a saprophyte coccus like *Gemella morbillorum* caused liver septic invasion, possibly through the portal venous system. US, CT, and MRI were unable to differentiate between septic or neoplastic abscess and the diagnosis was performed through percutaneous needle biopsy. The only possible source of the infection was the multiple colon diverticula. In addition, intermittent needle aspiration of the hepatic lesion -coupled with systemic antibiotic treatment- induced the complete resolution of the septic lesions and probably shortened the recovery time.
